# Genetic Susceptibility, Colony Size, and Water Temperature Drive White-Pox Disease on the Coral *Acropora palmata*

**DOI:** 10.1371/journal.pone.0110759

**Published:** 2014-11-05

**Authors:** Erinn M. Muller, Robert van Woesik

**Affiliations:** 1 Mote Marine Laboratory, Sarasota, Florida, United States of America; 2 Department of Biological Sciences, Florida Institute of Technology, Melbourne, Florida, United States of America; University of Genova, Italy

## Abstract

Outbreaks of coral diseases are one of the greatest threats to reef corals in the Caribbean, yet the mechanisms that lead to coral diseases are still largely unknown. Here we examined the spatial-temporal dynamics of white-pox disease on *Acropora palmata* coral colonies of known genotypes. We took a Bayesian approach, using Integrated Nested Laplace Approximation algorithms, to examine which covariates influenced the presence of white-pox disease over seven years. We showed that colony size, genetic susceptibility of the coral host, and high-water temperatures were the primary tested variables that were positively associated with the presence of white-pox disease on *A. palmata* colonies. Our study also showed that neither distance from previously diseased individuals, nor colony location, influenced the dynamics of white-pox disease. These results suggest that white-pox disease was most likely a consequence of anomalously high water temperatures that selectively compromised the oldest colonies and the most susceptible coral genotypes.

## Introduction

Infectious diseases are a major cause of coral decline worldwide, and are one of the main reasons that two Caribbean corals, *Acropora palmata* (Lamarck, 1816) and *A. cervicornis* (Lamarck, 1816), are now listed as threatened under the US Endangered Species Act [1]. Although outbreaks of coral disease have occurred since at least the 1970s in the Caribbean [2], researchers are still trying to determine which coral diseases are infectious, and whether the infectious diseases are also contagious [3]. Understanding whether coral diseases are contagious or not can help elucidate the mechanisms that drive disease activity on contemporary reefs.

An infectious disease is generally caused by micro-organisms such as bacteria, protozoans, fungi, or viruses, which enter organisms, survive, multiply [4], and cause negative physiological changes within the infected organisms. A contagious disease is one that is communicable by contact with, or through, some secretion from the infected individual [4]. Several studies have determined that many coral diseases are caused by microbial agents. In fact, much of the research within the past several decades has focused on identifying putative pathogens [5], [6], [7], [8], [9]. Still, it is unclear whether most coral diseases are indeed contagious. Determining whether a disease is contagious will provide critical information that is necessary to reduce disease outbreaks. For example, if a disease outbreak is caused by a novel pathogen that passes from individual to individual (i.e., it is contagious) then controlling the pathogenic source will reduce disease impacts. However, if a disease outbreak is primarily a result of an environmental stress on the population, then steps need to be taken to reduce that stress.

Over the last 20 years white-pox disease has caused considerable coral mortality on Caribbean reefs [6]. White-pox disease was first documented in 1996 on Eastern Dry Rocks Reef off Key West, Florida [10], and was considered to be exclusive to *A. palmata* [6]. Subsequent studies showed that white-pox disease affected *A. palmata* populations throughout the Caribbean [11]. Indeed, white-pox disease was reportedly responsible for approximately an 85% decline in *A. palmata*, between 1996 and 1998, on reefs throughout the Florida Keys [6].

White-pox disease is believed to be caused by an infectious agent. In 2002 Patterson and colleagues satisfied Koch's postulates and linked white-pox disease with the bacteria *Serratia marcescens* (Bizio, 1823) [6]. *S marcescens* is a gram-negative motile bacterium that is commonly found within the gut of many vertebrates, including humans, although it can also exist as a free-living microbe in soil and in seawater [12]. Yet *S. marcescens*, the putative pathogen, was not consistently found in corals showing signs of white-pox disease in the Florida Keys [13], nor were the bacteria found in diseased samples from St. John, US Virgin Islands [14]. Additionally, Lesser and Jarrett did not detect *S. marcescens* in either colonies of *A. palmata* that showed signs of white-pox disease, or in healthy-appearing colonies of *A. palmata* in the Bahamas [15]. *S. marcescens* was, however, found within the tissue of 'healthy' appearing coral colonies in St. John [14]. These conflicting results indicate that *S. marcescens* might not be the only causative agent of white-pox disease, or the bacteria might be only pathogenic under certain environmental conditions.

Previous studies have suggested that white-pox disease is also contagious. Field surveys from reefs in the Florida Keys showed colonies with white-pox disease were clustered, which suggests that the disease is contagious [6]. However, the spatial analyses of these surveys did not account for the naturally clustered distribution of coral colonies of *A. palmata* within their sites. Furthermore, colony fragmentation within populations is a common mode of asexual reproductionaaAz within corals [16]. Since asexual fragmentation is the most dominant mode of reproduction in *A. palmata*, clones on reefs are close, often adjacent, and therefore nearest neighbors are frequently the same genotype [17]. For example, in the Florida Keys, USA, patch reefs contain several colonies of *A. palmata*, but most colonies are of the same genotype [18]. Of the twenty *A. palmata* colonies collected on both Horseshoe Reef and Little Grecian Reef by Baums and colleagues, only one genotype was detected on each reef [18]. On the land [19] and in the oceans [20] susceptibility to disease varies among genotypes. Therefore, without knowing the distribution of the coral genotypes, the resultant clustering patterns, particularly in places such as the Florida Keys where cloning is high, may be a reflection of the genotypic susceptibility of clones, rather than a reflection of the spatial pattern of a contagious disease.

In addition to determining whether a disease is contagious or not, understanding the environmental conditions that foster disease outbreaks is critical. Two of the environmental factors that are known to influence the dynamics of coral disease are water temperature and irradiance [21], [22], [23], [24]. Temperature anomalies have been positively associated with outbreaks of white syndrome in the Great Barrier Reef [25]. Furthermore, coral bleaching caused by high water temperatures increases the likelihood of disease activity in the Caribbean as well as in the Pacific Ocean [10], [26], [27], and most likely also reduces the innate immune system of corals [28], [29], [47]. Elevated levels of irradiance also increase the severity of some coral diseases [30] and can lead to compromised coral hosts [31].

Another factor that may influence disease susceptibility is colony size. A large colony has a larger surface area than a small colony, which could translate to a higher ‘target’ area for pathogenic infections. Additionally, the size of a coral colony may be an indication of colony age, although fragmentation events can create small colonies that may be very old [19], [32]. Large colonies, however, are also likely to be long-lived individuals and could possibly suffer from senescence [33]. The prevalence of white-pox disease tends to increase with colony size, but whether an increase in prevalence is a consequence of an increased ‘target’ area for pathogens, or the result of senescence is currently unknown [34]. Determining whether white-pox disease is contagious (i.e., influenced by spatial location in relation to other infected individuals) will provide insight into whether ‘target’ area or senescence causes large colonies to be more susceptible to disease infection than small colonies.

We examined the dynamics of white-pox disease on 69 *A. palmata* colonies in the US Virgin Islands (USVI) over seven years. The goals of this study were to: (i) use a space-time Bayesian model to determine whether spatial and temporal patterns of white-pox disease were indicative of a contagious disease that was potentially transmitted to nearest neighbors, and (ii) test a suite of covariates that might influence disease activity. We were particularly interested in the occurrence of reinfections of particular genotypes, whether colony size played a role in infection, and to what extent irradiance and water temperature affected the prevalence of white-pox disease.

## Materials and Methods

### Field surveys

Haulover Bay is located on the northeast side of St. John, US Virgin Islands. This bay supports a fringing reef adjacent to the shoreline. The reef is populated with isolated colonies of *A. palmata*, between 1 and 3 m depth. In February 2003, every colony of *A. palmata* within the west side of Haulover Bay was identified, photographed, and tagged, for a total of 69 individual colonies. Each colony was also given a Global Positioning System (GPS) waypoint. These colonies were then monitored every month for the next 7 years, from February 2003 to December 2009, for the presence or absence of white-pox disease [34] (Table S1). A previous study examined the genotypes of 48 of the 69 corals found in Haulover Bay [35]. Out of the 48 coral colonies analyzed, 43 colonies were genetically distinct [35]. Therefore, in the present study, any detection of spatial clustering patterns of white-pox disease within Haulover Bay, St. John (USVI) would be a result of contagious disease transmission, rather than a result of genetic susceptibility of clones.

### Environmental parameters

Water temperature data was collected using a Hobo Temperature Pro v2 data logger, attached to the substrate, which recorded temperature every 10 minutes. Temperature data used in the model were the average water temperature recorded for the 30 days prior to the field survey. Approximations of solar insolation (300-5000 nm), measured as kW m^−2^ day^−1^, were obtained for Haulover Bay from the trigonometric polynomial approximations, which calculated average monthly values on a 1°×1° coarse grid [36]. The equation was formulated from data made available by the National Aeronautics and Space Administration (NASA) Langley Research Center (LaRC) Atmospheric Science Data Center Surface meteorological and Solar Energy (SSE) 6.0 web portal supported by the NASA LaRC Prediction of Worldwide Energy Resource (POWER) Project (http://eosweb.larc.nasa.gov/sse/).

### The model

We took a Bayesian space-time modeling approach adapted from Cameletti and colleagues to analyze the presence or absence of white-pox disease on the monitored 69 colonies of *A. palmata* [37] (Text S1). We let *y*(*s_i_,t*) represent the realization of the spatio-temporal binomial process *Y*(·,·), which denotes the presence or absence of white-pox disease at colony *i*  =  1,…,*d*, located at *s_i_* and day *t* = 1,…,*T*. We assumed that *y*(*s_i_,t*)  =  *z*(*s_i_,t*) *β* + ξ(*s_i_,t)* + ε(*s_i_,t*)

where z(*s_i_,t*) is [*z_1_*(*s_i_,t*),…*z_p_*(*s_i_,t*)] that represents the vector of *p* covariates for colony location *s_i_* at time *t. β* is (*β_1_*,…,*β_p_*), the coefficient vector. ξ(*s_i_,t*) is the realization of the state process, which is the unobserved level of disease occurrence that is assumed to be a spatio-temporal Gaussian field that changes over time with first order autoregressive dynamics. ε(*s_i_,t*) is the measurement error defined by a Gaussian white-noise process (*∼ N* (*0, σ^2^_ε_*). We used the specified model with a binomial response variable [37]. The output of the model provides the mean, the standard deviation, the 2.5% and 97.5% quantiles, and the mode for the correlation coefficients of each covariate. Significant values are those with 2.5% and 97.5% quantile ranges that do not span zero. Positive and negative values depict the direction of the association.

Our approach used a Gaussian Markov Random Field (GMRF) function, which is a spatial process that models the spatial dependence of data observed on geographic regions [38]. The GMRF computational properties were enhanced by using Integrated Nested Laplace Approximations (INLA) [39] for Bayesian inference. INLA is a computationally effective algorithm that produces fast and accurate approximations of posterior distributions [37]. All analyses were conducted using R version 3.0.1 [40] and the INLA package (www.r-inla.org; see Text S1).

**Figure 1:**
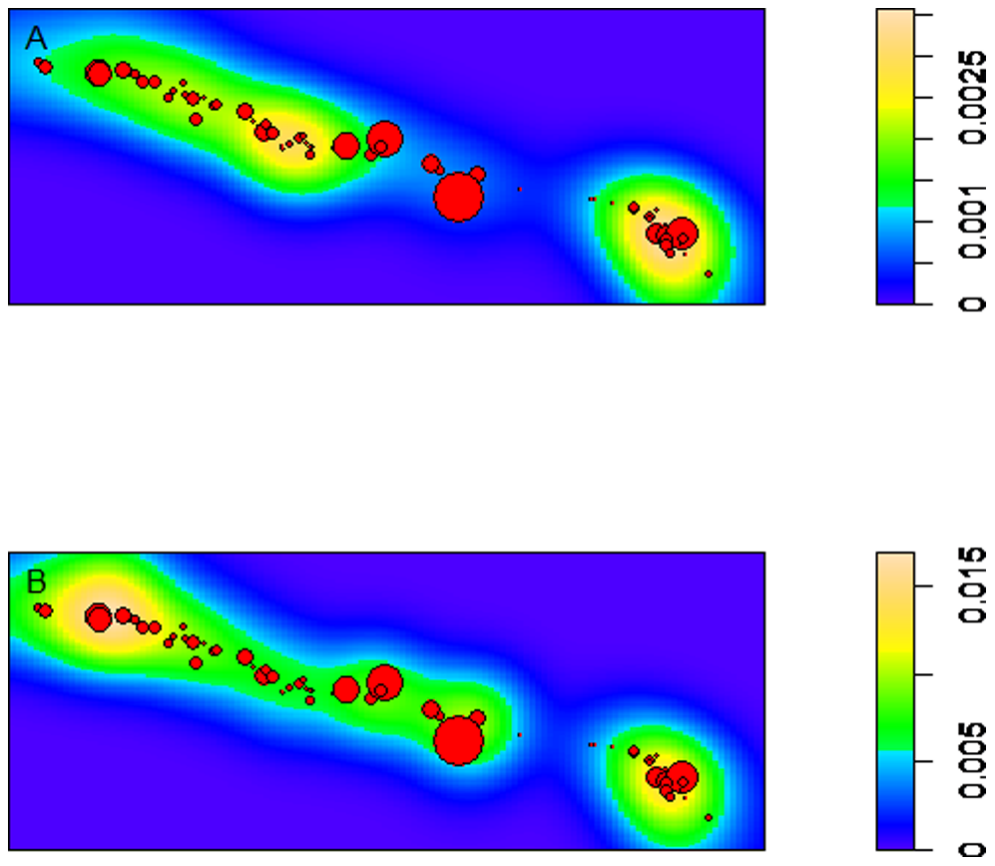
Density plots of colonies of *Acropora palmata* at Haulover Bay in St. John, United States Virgin Islands (USVI). Density plot in A) is based on the expected number of random points per unit area (i.e. colony density), where warm colors (yellow) denote dense areas of individual colonies, whereas cool colors indicate locations sparse of coral colonies. The density plot in B) represents the intensity of white-pox disease reoccurrence within the spatial plane of annual disease activity during the 7-year study period, from February 2003 to December 2009. The axes of the spatial area are 220 m by 560 m. The red dots represent the locations of individual coral colonies. The size of the red dots represents initial colony size recorded in February 2003. Density plots were created using the *density* function of the R package ‘spatstat’.

For the last decade, Markov Chain Monte Carlo (MCMC) techniques have been used in Bayesian analysis to predict the posterior marginal distribution. We note that INLA is a recent alternative to MCMC techniques in spatial-temporal modelling to predict the posterior marginal distribution. INLA techniques combine Gaussian Field with Matérn covariance functions to produce GMRFs by using stochastic partial differential equations (SPDE). This process speeds up the estimates and accuracy, and INLA does not have the same convergence problems as MCMC techniques. The SPDE approach also uses a finite element representation to define the Matérn field by triangulation of the domain. This approach is appropriate for our data, which were taken at irregular discrete locations on a coral reef (Fig 1).

Eight covariates were tested to determine whether they had a significant association with the presence or absence of white-pox disease on individual colonies through time (1). These covariates included: the spatial location of each colony measured as (i) the easting, and (ii) the northing locations (as georeferenced Universal Transverse Mercator (UTM) units), (iii) colony size (in cm^3^), (iv) the number of previous incidences of white-pox disease for each colony (we note that, although some colonies may have had a long history of disease before our study commenced, we started every colony at zero at the commencement of our study), (v) the distance to the nearest neighboring colony, (vi) the distance from a previously infected colony, (vii) water temperature, and (viii) solar insolation. Because water temperature and solar insolation vary on scales larger than the size of Haulover Bay [41], these covariate values were the same for all colonies within each time step, but varied for each time step. To illustrate spatial patterns in coral colony density and intensity of disease activity, a kernel smoothed intensity function was applied and plotted to the point pattern spatial data using the *spatstat* package in R [42].

## Results

Three of the tested covariates significantly influenced the presence or absence of disease on individual coral colonies. Colony size, number of previous infections, and water temperature all showed a significant positive association with white-pox disease presence (Table 1). There was no significant effect on disease activity of colony location variables (easting or northing), or distance to the closest colony. Additionally, the distance to a previously infected colony did not affect disease presence either. The mean correlation coefficients indicate that previous incidences of disease had the strongest correlation (0.93), followed by water temperature (0.43), and colony size (0.30). The density plot of disease incidences over the 7-year period showed that areas of high disease activity, particularly in Haulover Bay's northwest region, did not coincide with high densities of coral colonies (Fig 1A, 1B). In addition, the level of solar insolation did not affect the activity of white-pox disease.

**Table 1:** Posterior estimates of the covariate coefficient vector *β* of the linear model (Eq. 1) testing the effects of multiple covariates on the presence of white-pox disease on coral colonies of *Acropora palmata* monitored monthly from February 2003 to December 2009.

Fixed effects	Mean	Standard deviation	2.5% quantile	50% quantile	97.5% quantile	Mode
Intercept	−3.29	0.25	−3.79	−3.29	−2.83	−3.28
Northing	0.29	0.83	−1.32	0.29	1.89	0.28
Easting	0.44	0.81	−1.14	0.43	2.05	0.42
**Colony size**	**0.30**	**0.09**	**0.12**	**0.30**	**0.47**	**0.30**
**Previous incidences**	**0.93**	**0.13**	**0.68**	**0.93**	**1.17**	**0.94**
Distance	−0.13	0.14	−0.41	−0.12	0.14	−0.12
Previous distance	−0.07	0.06	−0.19	−0.07	0.04	−0.07
**Water temperature**	**0.43**	**0.07**	**0.29**	**0.43**	**0.58**	**0.43**
Solar insolation	0.02	0.07	−0.12	0.02	0.16	0.01

The 8 covariates were: (i) easting; (ii) northing; (iii) colony size; (iv) previous incidences of disease; (v) distance to nearest neighbor; (vi) distance to previously infected colony; (vii) water temperature; and (viii) solar insolation. Significant covariates are those with correlation coefficients that do not cross 0 within the 2.5 and 97.5% quantile. Rows that are bold indicate significant differences. Positive and negative values represent the directional relationship between the covariate and disease presence.

## Discussion

The results of the model indicated that the spatial location of a particular coral colony did not significantly influence the probability of a colony manifesting white-pox disease. Additionally, the distance to nearest neighbors, and the distance to colonies that were previously infected with white-pox disease also had no significant influence on disease presence or absence. If white-pox disease is in fact contagious, then the colonies neighboring the infected colonies should be at a greater risk of obtaining the disease than more distant colonies, and spatial clustering would have occurred. The results did not show a neighborhood effect. Therefore, white-pox disease is most likely not a contagious disease *in situ*, showing no form of dependency on colony density.

Colony size significantly influenced the activity of white-pox disease. Compared with small-sized corals, large-sized colonies were more likely to show signs of white-pox disease. These results may be a reflection of an infectious disease that is a function of the available ‘target’ area, or a result of colony senescence [34]. Several other coral diseases, including ulcerative white-spot disease on massive *Porites* spp. and white-plague disease [43], [44], are more common on large-sized colonies, but the direct mechanism is still unclear. Since the distance from a previously infected colony did not influence white-pox disease occurrence, at t_+1_ (i.e., in the following month), then the positive association between colony size and disease activity was not likely to be a consequence of simply a larger target area. Our results instead suggest that large colonies are more likely to be susceptible to white-pox because they are older, with potentially reduced defense mechanisms because of senescence, than small colonies.

The number of previous infections that each *A. palmata* colony had experienced also significantly affected whether disease occurred on colonies within a given time step. Therefore, the more disease incidences a colony had experienced, the more likely that disease would again appear. There are several possibilities that may explain why the number of previous infections may have affected the probability of reinfection. One possibility may be that once a colony is infected with a pathogen, the infectious agent may reside within the organism and an environmental cue may reinitiate the manifestation of disease signs. Colonies that are initially infected may also become more susceptible over time. An alternative explanation is that colonies that acquire disease may be genetically more predisposed to the particular infectious agent that causes white-pox disease on *A. palmata*. Indeed, the identified pathogen, *S. marcescens*, has been found within healthy colonies more often than in diseased colonies at Haulover Bay, St. John (USVI) [14]. These results suggest that *S. marcescens* may be a regular component of the *A. palmata* microbiome within Haulover Bay, and that some coral genotypes might be more innately susceptible to the pathogenicity of this infectious agent than other coral genotypes, especially under stressful environmental conditions.

Previous studies have shown that some coral genotypes are resistant to disease infections [45], but their percentages were low (∼6%). Susceptibility, most likely, follows a continuum, where few individuals are resistant and few individuals are highly susceptible to disease. Although more experimental work is needed, our results suggest that there are several genotypes of *A. palmata* within Haulover Bay that are highly susceptible to white-pox disease. This information, combined with previous studies that showed that the putative pathogen is a common component of both diseased and non-diseased *A. palmata* colonies in Haulover Bay [14], indicate that the manifestation of white-pox disease is most likely a consequence of genetic susceptibility to environmental stress, rather than a consequence of repeated, novel infections.

Environmental conditions that are known to influence the prevalence of white-pox disease include high water temperature [21]. The present study showed that high temperatures were highly correlated with, and most likely influenced by, white-pox disease on *A. palmata* at Haulover Bay. Solar insolation, however, was not a significant covariate of disease (Table 1). The positive association between white-pox disease and temperature has been previously documented within Haulover Bay [35], on neighboring reefs in St. John [21], and in the Florida Keys [6]. White-pox disease has a seasonal cycle; prevalence tends to increase during months of high sea-surface temperature [35]. Several studies suggest that the positive association between disease prevalence on corals and water temperature is most likely linked to host susceptibility, rather than to pathogenic virulence [21], [46]. Indeed, high water temperature causes stress to coral colonies, often making them more susceptible to disease infection [25], [28], [47], [48], [49].

Our study showed that the presence of white-pox disease on *A. palmata* was a combination of high water temperatures and the genetic susceptibility of the host. Furthermore, white-pox disease did not appear to be contagious *in situ*. The pathogens that cause these specific signs of disease are, therefore, likely to be a common component of the coral's microbiome, but they only elicit signs of disease when found on susceptible coral hosts, and only when environmental conditions favor disease activity. Our spatial analysis showed that colony location had no influence on the presence or absence of white-pox disease. Therefore, future resilience of *A. palmata* to white-pox disease relies on (i) the survivability of specific coral genomes and (ii) the proliferation of disease resistant corals under strong selective pressure by the environment.

## Supporting Information

Table S1 **Disease and environmental data used within the Bayesian space-time model.**
(CSV)

Text S1 **R code for the Bayesian space-time model, adapted from Camelleti and colleagues [35].**
(DOCX)
