# Supplementary material for: Genetic Susceptibility, Colony Size, and Water Temperature Drive White-Pox Disease on the Coral Acropora palmata
Source: PLoS One. 2014 Nov 5;9(11):e110759. doi: 10.1371/journal.pone.0110759 (PMC4220941; doi:10.1371/journal.pone.0110759)
Supplement: Text S1 — R code for the Bayesian space-time model, adapted from Camelleti and colleagues [35]. (DOCX) [file pone.0110759.s002.docx]

**Text S1**

Supporting Information 1: R code for the space-time model, modified from Cameletti et al. (2012)

##################################

## Using the SPDE approach and the INLA algorithm for spatio-temporal

## modelling and mapping

##

## updated from: M.Cameletti, F.Lindgren, D.Simpson, H.Rue (2012)

##################################

require(INLA)

inla.update(testing=TRUE)

require(fields)

require(abind)

setwd("C:/Users/emuller/Documents/Mote/R teaching files/INLA/Cameletti et al 2012")

## ################################

## Load the data

## ################################

Haulover_data <-read.table("Supporting Table 1",header=TRUE,sep=",")

coordinates <-read.table("haulover_coordinates.csv",header=TRUE,sep=",")

rownames(coordinates) = coordinates[,"Colony.ID"]

i_day = 1

which_date = unique(Haulover_data$Date)[i_day]

print(paste("**---- You will get a prediction for ", which_date, "---**"))

## ################################

## Work out how many colonies and days there are

## ################################

n_colonies <- length(coordinates$Colony.ID)

n_data <- length(Haulover_data$Colony.ID)

n_days <- as.integer(n_data/n_colonies)

##--- Check that the data is OK

if (n_data %% n_colonies != 0) {

print("The number of data points needs to be an integer multiple of the number of colonies!")

return

}

## ################################

##Standardize covariates

## ################################

##--- The covariates are standardised using the mean and std.dev.

mean_covariates = apply(Haulover_data[,3:10],2,mean)

sd_covariates = apply(Haulover_data[,3:10],2,sd)

Haulover_data[,3:10] =

scale(Haulover_data[,3:10],

mean_covariates, sd_covariates)

Haulover_data$time = rep(1:n_days,each = n_colonies)

## ################################

## Estimation

## ################################

## ################################

## Triangulation using borders

## ################################

mesh =

inla.mesh.create.helper(points=cbind(coordinates$Northing,

coordinates$Easting),

offset=c(10, 140),

max.edge=c(50, 1000),

min.angle=c(26, 21),

cutoff=0,

plot.delay=NULL

)

##--- Plot the triangulation

plot(mesh)

points(coordinates$Northing, coordinates$Easting, pch=20, cex=2, col=2)

## ################################

## Make the SPDE object and the formula

## ################################

##--- Construct the SPDE object

spde = inla.spde2.matern(mesh=mesh, alpha=2)

##--- Observation structure for estimation data

A.est =

inla.spde.make.A(mesh,

loc=

as.matrix(coordinates[Haulover_data$Colony.ID,

c("Northing","Easting")]),

group=Haulover_data$time,

n.group=n_days

)

##--- Observation structure for field prediction

A.pred =

inla.spde.make.A(mesh, group=i_day, n.group=n_days)

field.indices =

inla.spde.make.index("field",

n.mesh=mesh$n,

n.group=n_days)

stack.est =

inla.stack(data=list(DIS=Haulover_data$DIS),

A=list(A.est, 1),

effects=

list(c(field.indices,

list(Intercept=1)),

list(Haulover_data[,3:10])),

tag="est")

scaled.mesh.loc =

list(Northing=(rep(scale(mesh$loc[,1],

mean_covariates["Northing"],

sd_covariates["Northing"]),

n_days)),

Easting=(rep(scale(mesh$loc[,2],

mean_covariates["Easting"],

sd_covariates["Easting"]),

n_days)))

stack.pred =

inla.stack(data=list(DIS=NA),

A=list(A.pred),

effects=

list(c(field.indices,

scaled.mesh.loc,

list(Intercept=1)

)),

tag="pred")

stack = inla.stack(stack.est, stack.pred)

formula <- (DIS ~ -1 + Intercept + Northing + Easting + PIC + DIST + DFPIC + TEMP + IRR + SIZE + f(field, model=spde, group=field.group, control.group=list(model="ar1")))

## ################################

## Call INLA and get results

## ################################

result =

inla(formula,

data=inla.stack.data(stack, spde=spde),

family="binomial",

control.inla = list(reordering = "metis"),control.predictor=list(A=inla.stack.A(stack), compute=TRUE),

control.compute=list(cpo=TRUE),

keep=FALSE, verbose=FALSE)

print(summary(result))
